# Association Between Blood Urea Nitrogen and Delirium in Critically Ill Elderly Patients Without Kidney Diseases: A Retrospective Study and Mendelian Randomization Analysis

**DOI:** 10.1111/cns.70201

**Published:** 2025-01-03

**Authors:** Yipeng Fang, Xiaohong Tang, Ying Gao, Hui Xie, Yuehao Shen, Min Peng, Jie Liu, Yunfei Zhang, Yan Cui, Keliang Xie

**Affiliations:** ^1^ Department of Critical Care Medicine Tianjin Medical University General Hospital Tianjin China; ^2^ Firth Clinical College, Xinxiang Medical University Xinxiang China; ^3^ Tianjin Hospital of Tianjin University Tianjin China; ^4^ Department of Pathogen Biology, School of Basic Medical Sciences Tianjin Medical University Tianjin China; ^5^ Department of Anesthesiology Tianjin Institute of Anesthesiology, Tianjin Medical University General Hospital Tianjin China

**Keywords:** delirium, elderly, encephalopathy, ICU‐CAM, mendelian randomization, MIMIC IV, urea nitrogen

## Abstract

**Objective:**

This study investigates the association between blood urea nitrogen (BUN) levels and the risk of delirium in critically ill elderly patients without kidney disease.

**Methods:**

A retrospective analysis was conducted using data from the MIMIC‐IV database. The relationship between BUN and delirium risk was illustrated through the restricted cubic spline (RCS) method. Patients were stratified by BUN levels and examined for delirium incidence using logistic regression, subgroup analysis, and sensitivity analysis. Mendelian randomization (MR) was employed to explore potential causal relationships.

**Results:**

The maximum BUN level exhibited the strongest non‐linear positive association with the odds of delirium. Compared to the Q1 group, both the Q3 (23–31 mg/dL) and Q4 (> 31 mg/dL) groups consistently showed increased odds of developing delirium. Subgroup analysis identified a significant interaction between BUN and midazolam, with the association only evident in patients not exposed to midazolam. After adjusting for baseline characteristics, the odds ratio for delirium was found to range from 1.35 to 1.39 in those with BUN levels exceeding 23 mg/dL. However, the MR analysis did not reveal any significant causal relationship (all *p* > 0.05).

**Conclusion:**

In conclusion, elevated BUN may be a risk factor for delirium in critically ill elderly patients without renal dysfunction, with this potential link possibly modulated by midazolam exposure.

## Graphical abstract

This retrospective study found a nonlinear positive association between the levels of maximum blood urea nitrogen (BUN) and the odds of delirium in critically ill elderly patients without kidney diseases. The optimal cut‐off value of maximum BUN was 23 mg/dL. A significant interaction between BUN and midazolam, with the association only evident in patients not exposed to midazolam when utilizing BUN for this purpose, it is critical to account for the potential confounding effect of midazolam.

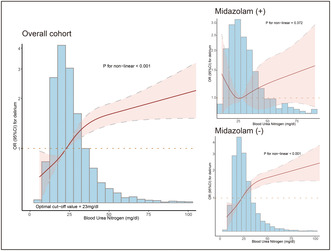

## 1 Background

Delirium is an acute, fluctuating neuropsychiatric disorder characterized by altered consciousness, cognitive impairment, memory loss, disorientation, and attention deficits [1]. It frequently complicates the care of elderly patients, particularly those in critical condition. Delirium incidence among elderly patients ranges from 14% to 56% in general hospital wards and 70% to 87% in intensive care units (ICUs), far exceeding the rates observed in the broader hospitalized population (14% to 24%) [2]. This condition leads to higher healthcare costs, prolonged mechanical ventilation, extended hospitalizations, increased mortality, and long‐term cognitive decline, including dementia [3, 4]. Monthly healthcare expenses for critically ill patients with delirium can reach up to $17,838, creating a significant social and economic burden [5]. Due to the lack of effective treatments, early identification of risk factors and preventive strategies is essential for improving patient outcomes [6]. Diagnosing delirium in elderly patients is particularly challenging, often going unnoticed due to multiple comorbidities and the complex etiology of the condition [7, 8]. This diagnostic delay can exacerbate the severity of the illness and worsen prognosis [7]. As a result, research on risk factors for delirium in elderly patients remains vital, offering key insights for early detection and intervention.

Blood urea nitrogen (BUN) is a widely recognized biomarker for assessing kidney function in clinical practice. In recent years, multiple studies have established elevated BUN levels as an independent risk factor for delirium occurrence [9, 10, 11]. For each 1 mmol/L increase in BUN upon admission, the risk of delirium rises by 18% (OR 1.018, 95% CI 1.006–1.031) [10], and BUN levels exceeding 20 mg/dL have been shown to increase the delirium risk by 58% (OR 1.58, 95% CI 1.06–2.36) [9]. Since elevated BUN is closely linked to impaired renal function, its predictive value for delirium is primarily related to kidney injury. Acute kidney injury (AKI) is a significant risk factor for delirium development, with Zipser et al. reporting a ninefold increased risk of delirium in patients with AKI [12]. The severity of AKI is also positively correlated with delirium risk, with patients in Stage 2 and Stage 3 AKI having a notably higher incidence compared to those in Stage 1 [13, 14]. Moreover, end‐stage renal failure has been confirmed to strongly correlate with delirium onset [4, 15]. However, it remains unclear whether elevated BUN levels in the absence of renal impairment are similarly associated with an increased risk of delirium and whether BUN may influence delirium development via non‐renal pathways.

This study aims to investigate the potential link between BUN levels and the risk of delirium in critically ill elderly patients without renal impairment, with the objective of providing new insights for the early assessment and management of delirium in this patient population.

## 2 Materials and Methods

### 2.1 Data Sources and Study Design

Data for this study were derived from the Medical Information Mart for Intensive Care IV (MIMIC IV) database [16], which aggregates clinical data from over 190,000 patients and 450,000 hospital admissions at the Beth Israel Deaconess Medical Center between 2008 and 2019. Access to MIMIC IV was granted following the completion of a web‐based course and examination administered by the National Institutes of Health. This manuscript adheres to the Strengthening the Reporting of Observational Studies in Epidemiology (STROBE) guidelines [17].

### 2.2 Population Selection

The inclusion criteria targeted elderly patients (age ≥ 65 years) admitted to the ICU, with only the first ICU admission record retained for individuals with multiple admissions. Patients with AKI, chronic kidney disease (CKD), or those undergoing renal replacement therapy (RRT) were excluded. CKD was identified using International Classification of Diseases (ICD) codes and the Charlson score (see Table S1), and AKI was defined in accordance with the Kidney Disease Improving Global Outcomes (KDIGO) creatinine standards [18]. Patients were also excluded if they had (1) gastrointestinal bleeding, (2) incomplete BUN records during their ICU stay, or (3) ICU stays of ≤ 48 h.

### 2.3 Exposure and Endpoint

Serum BUN levels were the exposure of interest, with initial, maximum, and average BUN levels recorded during the ICU stay. Differences in BUN levels, baseline characteristics, and clinical outcomes between the delirium and non‐delirium groups were analyzed. Additionally, patients were categorized into quartiles based on their highest BUN levels, and outcomes were compared across these quartiles. Baseline variables included age, gender, race, weight, comorbidities (e.g., cerebral infarction, cerebral hemorrhage, coronary heart disease, heart failure, hypertension, diabetes mellitus, anemia, chronic pulmonary disease, liver disease, and malignant cancer, as detailed in Table S1), laboratory values at ICU admission (white blood cell count, hemoglobin, platelet count, sodium, potassium, creatinine), mean arterial pressure, ICU treatments (mechanical ventilation, vasoactive drugs, midazolam), and disease severity scores at admission, including the Sequential Organ Failure Assessment (SOFA) score and the Simplified Acute Physiological Score II (SAPS II). Current evidence suggests that the use of benzodiazepines (e.g., midazolam) is an independent risk factor for the occurrence of delirium, compared to propofol or dexmedetomidine [3, 19]. Therefore, the utilization of midazolam has been deemed as a crucial factor of baseline information in our research. The code for data extraction is available on GitHub (https://github.com/MIT‐LCP/mimic‐iv).

The primary outcome was the onset of delirium during the ICU stay, diagnosed using the Confusion Assessment Method for the Intensive Care Unit (CAM‐ICU) tools [20]. Secondary outcomes included 28‐day mortality, 90‐day mortality, hospital mortality, ICU mortality, length of hospital stay (hospital‐LOS), and length of ICU stay (ICU‐LOS).

### 2.4 Statistical Analysis

Patients were classified based on whether delirium occurred, and baseline characteristics and outcomes were compared between the delirium and non‐delirium groups. Additionally, patients were stratified into quartiles based on the interquartile range (IQR) of BUN: Q1 (BUN ≤ 18 mg/dL), Q2 (18 mg/dL < BUN ≤ 23 mg/dL), Q3 (23 mg/dL < BUN ≤ 31 mg/dL), and Q4 (BUN > 31 mg/dL). The distribution of continuous variables was assessed using the Shapiro–Wilk test (Table S2). Normally distributed continuous variables were expressed as mean ± standard deviation (SD) and analyzed using Student's T‐test or one‐way ANOVA, while non‐normally distributed variables were reported as median and IQR and analyzed using rank‐sum or Kruskal‐Wallis (K‐W) tests. Categorical variables were presented as counts and percentages and compared using the Chi‐square test. To control for multiple comparisons, the Bonferroni correction was applied to adjust *p*‐values.

**FIGURE 1:**
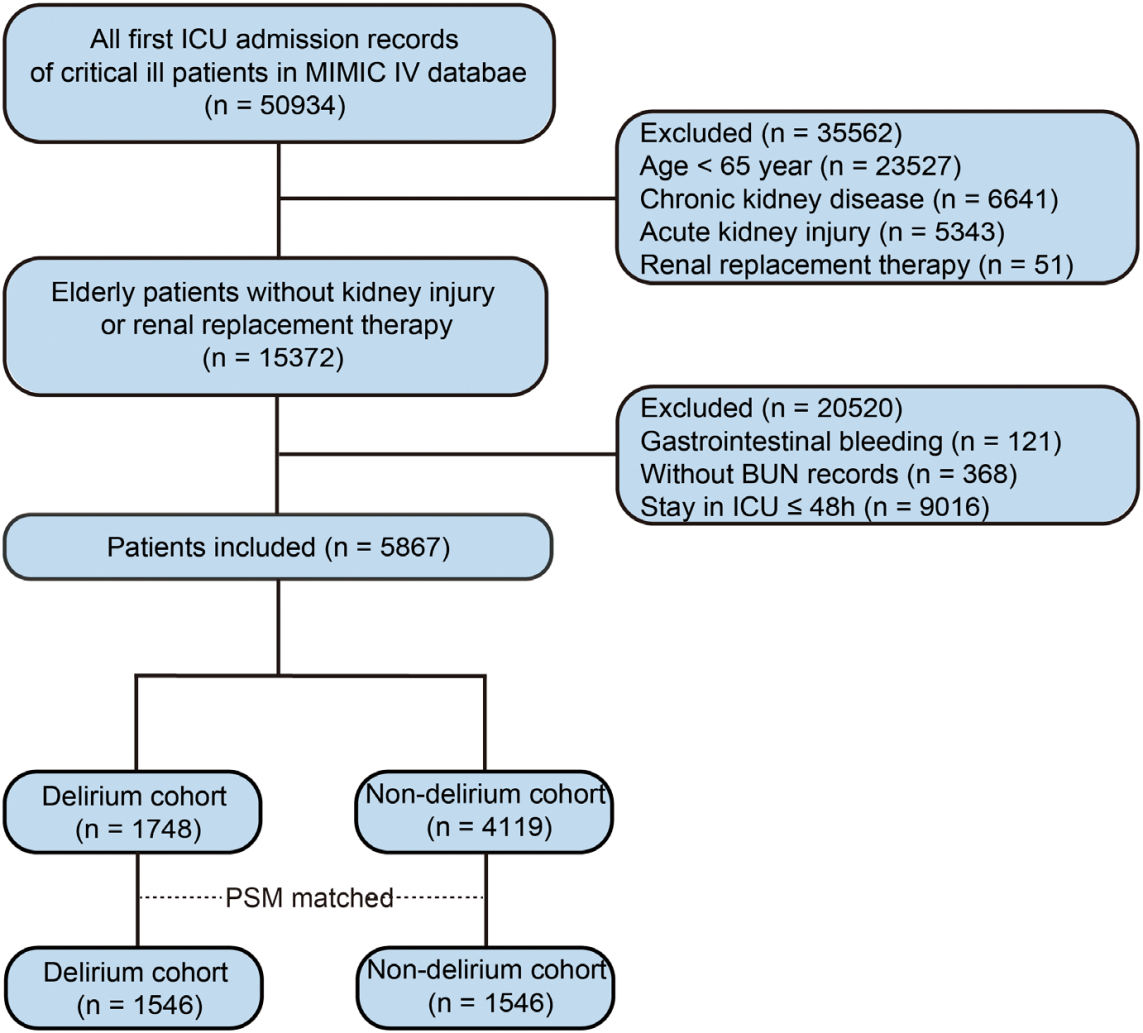
Flow chart for patient selection.

The relationship between BUN and the odds of delirium was visualized through a restricted cubic spline (RCS) curve with four standard knots at the 0.05, 0.35, 0.65, and 0.95 percentiles. Logistic regression analysis was conducted to further evaluate the predictive value of BUN for delirium, adjusting for potential confounders. Based on the Directed Acyclic Graphs (DAGs) theory, renal dysfunction (AKI and CKD) and gastrointestinal bleeding were identified as confounders that affect both the exposure (BUN) and clinical outcomes (delirium/mortality) [21]. To mitigate these effects, this study excluded patients with AKI, CKD, and gastrointestinal bleeding, focusing on exploring the relationship between BUN and the risk of delirium and mortality via non‐renal pathways. After excluding these patients, demographic factors, laboratory indicators, comorbidities, and specific interventions were included as potential confounders to balance illness severity and other factors contributing to delirium between the groups. The first adjusted model included demographic and comorbidity data. The second model expanded on this by incorporating additional laboratory parameters. The third model further included special interventions and disease severity scores. Variables with a variance inflation factor (VIF) > 10 were converted into binary variables to address multicollinearity. To control for confounding factors, propensity score matching (PSM) was employed for a balanced baseline in the sensitivity analysis [22]. Nearest neighbor matching with a caliper of 0.02 and a 1:1 matching ratio without replacement was used. Ultimately, 3092 patients were included in the PSM cohort, comprising 1546 patients with delirium and 1546 non‐delirium individuals (Figure 1). Subgroup analysis was performed to validate the robustness of the findings and identify potential interaction factors, stratifying by sex, race, comorbidities (e.g., sepsis, stroke, cerebral hemorrhage), midazolam exposure, vasopressor exposure, mechanical ventilation, and SOFA scores (median: 4).

The causal relationship between BUN and delirium was assessed using MR analysis [23]. Genome‐wide association study (GWAS) summary data for delirium (finngen_R9_F5_DELIRIUM) were obtained from the Finngen R9 database (https://r9.finngen.fi/) [24], while data for BUN (bbj‐a‐11) were sourced from the IEU GWAS project database (https://gwas.mrcieu.ac.uk/). A two‐sample MR analysis was conducted using the “TwoSampleMR” package with default parameters. IVs strongly correlated with the outcome phenotype (*p* < 5e−8) were excluded. The inverse variance weighted (IVW) method under random effects [25], the weighted median (WM) [26], and MR‐Egger regression [27] were employed for statistical analysis. Results were visualized through scatter plots, forest plots, leave‐one‐out sensitivity analysis plots, and funnel plots. Directional horizontal pleiotropy and heterogeneity were evaluated using MR‐Egger regression intercepts and Cochran's Q test.

All statistical analyses were conducted with Stata (version SE 15.0) and R (version 4.3.2), with a two‐sided *p‐*value < 0.05 considered statistically significant.

## 3 Results

### 3.1 Sample Size and Baseline Information

A total of 5867 patients were included in the study (Figure 1). Statistically significant differences were observed between the delirium and non‐delirium groups in terms of age, white ethnicity, and body weight (all *p* ≤ 0.001). Additionally, the proportions of patients with sepsis, cerebral infarction, cerebral hemorrhage, and anemia were higher in the delirium group, while the proportion of patients with malignant cancer was lower. The delirium group also exhibited significantly higher initial and maximum (25 [3, 33] vs. 23 [17, 29], *p* < 0.001) and average BUN levels during the ICU stay (all *p* < 0.01). Moreover, higher proportions of patients in the delirium group required mechanical ventilation, vasoactive drugs, and midazolam (all *p* < 0.01). This group also demonstrated higher SOFA and SAPS II scores (both *p* < 0.01). Mortality rates were significantly elevated in the delirium group, with higher hospital mortality (12.87% vs. 6.77%), ICU mortality, 28‐day mortality, and 90‐day mortality, alongside longer hospital‐LOS and ICU‐LOS (all *p* < 0.001, Table 1).

**TABLE 1:** Baseline information and clinical outcomes in elderly patients with sepsis with and without delirium.

Variable	Overall	Non‐delirium	Delirium	*p*
Number	5867	4119	1748	
Age (years)	76.6 (70.3, 84.0)	76.1 (69.9, 83.4)	77.7 (71.7, 85.7)	< 0.001
Male (%)	2862 (48.78)	2034 (49.38)	828 (47.37)	0.157
Ethnicity, white (%)	4004 (68.25)	2937 (71.30)	1067 (61.04)	< 0.001
Weight (kg)	75.0 (62.8, 87.2)	76.0 (63.8, 88.2)	76.7 (60.7, 85.2)	< 0.001
Comorbidity				
Sepsis (%)	2967 (50.57)	1864 (45.25)	1103 (63.10)	< 0.001
Cerebral infarction (%)	1010 (17.21)	611 (14.83)	399 (22.83)	< 0.001
Cerebral hemorrhage (%)	442 (7.53)	238 (5.78)	204 (11.67)	< 0.001
Coronary heart disease (%)	1324 (22.57)	949 (23.04)	375 (21.45)	0.184
Heart failure (%)	1471 (25.07)	1032 (25.05)	439 (25.11)	0.961
Hypertension (%)	3861 (65.81)	2729 (66.25)	1132 (64.76)	0.270
Diabetes mellitus (%)	1519 (25.89)	1041 (25.27)	478 (27.35)	0.097
Atrial fibrillation (%)	2211 (37.69)	1534 (37.24)	677 (38.73)	0.282
Anemia (%)	1260 (21.48)	849 (20.61)	411 (23.51)	0.013
Chronic pulmonary disease (%)	1575 (26.85)	1136 (27.58)	439 (25.11)	0.051
Liver disease (%)	289 (4.93)	198 (4.81)	91 (5.21)	0.518
Malignant cancer (%)	849 (14.47)	630 (15.29)	219 (12.53)	0.006
Laboratory parameter				
White blood cell (k/μL)	10.5 (7.8, 14.1)	10.3 (7.7, 13.9)	10.7 (8.1, 14.6)	< 0.001
Hemoglobin (g/dL)	11.1 (9.6, 12.5)	11.0 (9.6, 12.5)	11.1 (9.6, 12.5)	0.708
Platelets (k/μL)	192 (144, 251)	193 (145, 256)	188 (142, 240)	< 0.001
Sodium (mmol/L)	138 (136, 141)	138 (136, 141)	139 (139, 142)	< 0.001
Potassium (mmol/L)	4.1 (3.7, 4.5)	4.1 (3.7, 4.5)	4.0 (3.7, 4.4)	0.059
Creatinine (mg/dL)	0.9 (0.8, 1.2)	0.9 (0.8, 1.2)	0.9 (0.8, 1.2)	0.250
Mean blood pressure (mmHg)	78.2 (72.1, 85.4)	77.2 (71.4, 84.6)	80.3 (74.2, 87.3)	< 0.001
Intervention				
Mechanical ventilation (%)	2300 (39.20)	1374 (33.36)	926 (52.97)	< 0.001
Vasoactive drug (%)	1247 (21.25)	737 (17.89)	510 (29.18)	< 0.001
Midazolam exposure (%)	830 (14.15)	546 (13.26)	284 (16.25)	0.003
Disease severity score				
SOFA score	4 (3, 6)	4 (2, 6)	5 (4, 7)	< 0.001
SAPS II score	36 (30, 43)	35 (29, 42)	39 (33, 47)	< 0.001
Blood urea nitrogen (mg/dL)				
Initial value	18 (13, 24)	17 (13, 24)	18 (13, 25)	0.004
Maximum value	23 (18, 31)	23 (17, 30)	25 (19, 34)	< 0.001
Mean value	18 (14, 23)	17 (13, 23)	18 (14, 24)	< 0.001
Outcomes				
Hospital mortality (%)	504 (8.59)	279 (6.77)	225 (12.87)	< 0.001
ICU mortality (%)	283 (4.82)	161 (3.91)	122 (6.98)	< 0.001
28‐day mortality (%)	776 (13.23)	428 (10.39)	348 (19.91)	< 0.001
90‐day mortality (%)	1133 (19.31)	661 (16.05)	472 (27.00)	< 0.001
Hospital LOS (days)	7.8 (5.3, 12.0)	7.1 (5.0, 10.6)	9.5 (6.6, 15.6)	< 0.001
ICU LOS (days)	3.2 (2.5, 4.8)	3.0 (2.3, 4.2)	4.0 (2.9, 6.2)	< 0.001

Abbreviations: ICU, intensive care unit; LOS, length of stay; SAPS, Simplified Acute Physiological Score; SOFA, Sequential Organ Failure Assessment; Tip, Continuous variables are presented as median (first quartile–third quartile), and categorical variables are presented as count (percentage).

### 3.2 Visualization of the Non‐Linear Relationships by Restricted Cubic Spline

**FIGURE 2:**
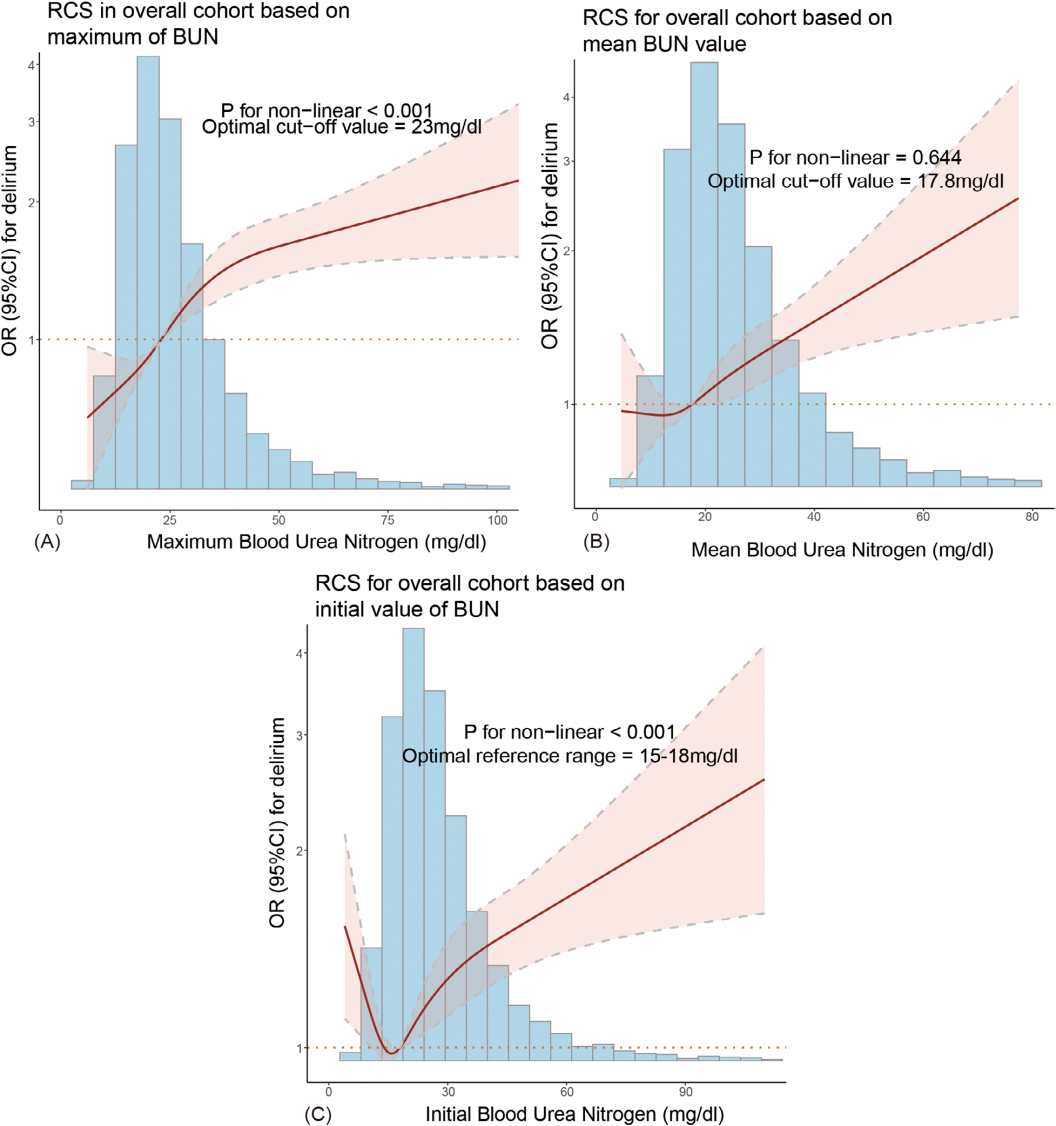
Restricted cubic spline of BUN and the risk of delirium in critically ill elderly patients. (A) Maximum; (B) Mean value; (C) Initial value.

The RCS curve illustrated the relationship between BUN levels and delirium odds. A nonlinear positive correlation was observed between maximum BUN levels and delirium development (*p* for nonlinearity < 0.001, Figure 2A), with delirium odds rising as maximum BUN increased. The optimal cut‐off value was 23 mg/dL. A linear positive association was identified between the mean BUN value and delirium odds, with an optimal cut‐off of 17.8 mg/dL (*p* for nonlinearity = 0.644, Figure 2B). However, a U‐shaped relationship was detected between initial BUN levels and delirium odds, with an optimal reference range of 15–18 mg/dL (*p* for nonlinearity < 0.001, Figure 2C).

### 3.3 Outcomes in Different BUN Categories

Patients were divided into quartiles (Q1–Q4) based on the IQR of BUN levels (Table 2 and Tables S3 and S4). Regarding maximum BUN categories, significant differences were observed between the quartiles in terms of delirium incidence, all mortality indicators, and LOS (all *p* < 0.01, Table 2). As BUN levels increased, the incidence of delirium also rose, with Q4 showing the highest delirium incidence (36.85%), followed by Q3 (32.29%). A similar pattern was noted across mortality indicators, hospital LOS, and ICU‐LOS.

**TABLE 2:** Clinical outcomes among patients in different BUN categories.

	Q1 (BUN ≤ 18 mg/dL)	Q2 (18 < BUN ≤ 23 mg/dL)	Q3 (23 < BUN ≤ 31 mg/dL)	Q4 (BUN > 31 mg/dL)	*p*
Number (%)	1660 (28.29)	1287 (21.94)	1471 (25.07)	1449 (24.70)	
Delirium (%)	397 (23.92)	342 (26.57)	475 (32.29)	534 (36.85)	< 0.001
28‐day mortality (%)	155 (9.34)	137 (10.64)	205 (13.94)	279 (19.25)	< 0.001
90‐day mortality (%)	226 (13.61)	192 (14.92)	283 (19.24)	432 (29.81)	< 0.001
Hospital mortality (%)	102 (6.14)	79 (6.14)	125 (8.50)	198 (13.66)	< 0.001
ICU mortality (%)	63 (3.80)	48 (3.73)	82 (5.57)	90 (6.21)	0.002
Hospital LOS (days)	6.7 (4.7, 9.6)	7.3 (5.2, 10.9)	8.1 (5.7, 12.7)	9.5 (6.6, 14.9)	< 0.001
ICU LOS (days)	3.0 (2.3, 4.0)	3.1 (2.4, 4.4)	3.4 (2.5, 5.1)	3.7 (2.8, 5.9)	< 0.001

*Note:* Continuous variables are presented as median (first quartile–third quartile), and categorical variables are presented as count (percentage).

Abbreviations: BUN, blood urea nitrogen; ICU, intensive care unit; LOS, length of stays.

In Table S3, patients in the Q4 group for mean BUN had the highest delirium incidence, followed by Q3, Q2, and Q1 (*p* = 0.002). Comparable trends were observed for mortality and LOS. In Table S4, patients in Q4 of initial BUN also had the highest delirium incidence, followed by Q3, while those in Q2 exhibited the lowest delirium incidence.

### 3.4 Predictive Value of BUN in Delirium Development Using Logistic Regression Analysis

As presented in Table 3, logistic regression analysis showed that for every 1 mg/dL increase in maximum BUN levels, the odds of developing delirium increased by 1%–2% (OR = 1.01–1.02, all *p* < 0.001). When using the Q1 category as a reference, the Q3 and Q4 categories were significantly associated with higher odds of delirium, increasing by 28%–52% (OR = 1.28–1.52, all *p* < 0.01) and 35%–86% (OR = 1.35–1.86, all *p* < 0.01), respectively. For 28‐day mortality, each 1 mg/dL increase in maximum BUN levels was associated with a 2%–3% rise in odds (OR = 1.02–1.03 across different models, all *p* < 0.001). The Q3 and Q4 categories showed significantly higher odds of 28‐day mortality, with an OR of 1.34–1.57 (*p* < 0.05) for Q3 and 1.80–2.32 (*p* < 0.001) for Q4.

**TABLE 3:** Logistic regression analysis detecting the predictive value of BUN for delirium and 28‐day mortality in critically ill elderly patients.

	Unadjusted		Model 1		Model 2		Model 3	
	OR (95% CI)	*p*	OR (95% CI)	*p*	OR (95% CI)	*p*	OR (95% CI)	*p*
Delirium								
BUN (mg/dL)	1.02 (1.01–1.02)	< 0.001	1.01 (1.01–1.01)	< 0.001	1.02 (1.01–1.02)	< 0.001	1.01 (1.01–1.02)	< 0.001
Q1 (BUN ≤ 18 mg/dL)	Reference							
Q2 (18 < BUN ≤ 23 mg/dL)	1.15 (0.97–1.36)	0.099	1.11 (0.93–1.32)	0.251	1.11 (0.93–1.32)	0.245	1.05 (0.87–1.25)	0.627
Q3 (23 < BUN ≤ 31 mg/dL)	1.52 (1.30–1.78)	< 0.001	1.44 (1.22–1.70)	< 0.001	1.44 (1.22–1.71)	< 0.001	1.28 (1.07–1.52)	0.006
Q4 (BUN ≥ 31 mg/dL)	1.86 (1.59–2.17)	< 0.001	1.59 (1.34–1.88)	< 0.001	1.60 (1.33–1.94)	< 0.001	1.35 (1.01–1.65)	0.003
28‐day mortality								
BUN (mg/dL)	1.02 (1.01–1.02)	< 0.001	1.02 (1.01–1.02)	< 0.001	1.03 (1.02–1.03)	< 0.001	1.02 (1.01–1.03)	< 0.001
Q1 (BUN ≤ 18 mg/dL)	Reference							
Q2 (18 < BUN ≤ 23 mg/dL)	1.16 (0.91–1.47)	0.239	1.10 (0.86–1.42)	0.443	1.13 (0.88–1.46)	0.338	1.06 (0.82–1.37)	0.668
Q3 (23 < BUN ≤ 31 mg/dL)	1.57 (1.26–1.96)	< 0.001	1.48 (1.17–1.87)	0.001	1.52 (1.20–1.93)	< 0.001	1.34 (1.05–1.70)	0.019
Q4 (BUN ≥ 31 mg/dL)	2.32 (1.88–2.86)	< 0.001	2.08 (1.65–2.62)	< 0.001	2.29 (1.77–2.95)	< 0.001	1.80 (1.39–2.34)	< 0.001

*Note:* Model 1: adjustments were made for sex, age, ethnicity, body weight, and comorbidities, including cerebral infarction, cerebral hemorrhage, sepsis, hypertension, coronary heart disease, heart failure, atrial fibrillation, diabetes, chronic pulmonary disease, liver disease, anemia, and malignant cancer. Model 2: built upon Model 1 by adjusting for additional laboratory parameters, such as serum white blood cells, hemoglobin, platelets, sodium, potassium, and creatinine levels. Model 3: built upon Model 2 by additionally adjusting for mechanical ventilation, vasoactive drugs, SOFA score, and SAPS II score.

Abbreviations: CI, confidence interval; OR, odds ratio; SAPS II, Simplified Acute Physiology Score‐2; SOFA, Sequential Organ Failure Assessment.

After adjusting for potential confounders in the multiple logistic regression analysis, no significant associations were found between the mean or initial BUN values and the odds of delirium (Tables S5 and S6). However, both the mean and initial BUN values remained significantly associated with the odds of 28‐day mortality (all *p* < 0.05).

These results suggest that maximum BUN levels have a stronger association with the odds of delirium compared to mean or initial BUN values. Therefore, further analyses were conducted based on the maximum BUN levels. Patients were categorized into high‐BUN (BUN > 23 mg/dL) and low‐BUN (BUN ≤ 23 mg/dL) groups according to the optimal cut‐off value of the maximum BUN levels identified by the RCS curve.

### 3.5 Subgroup Analysis

**FIGURE 3:**
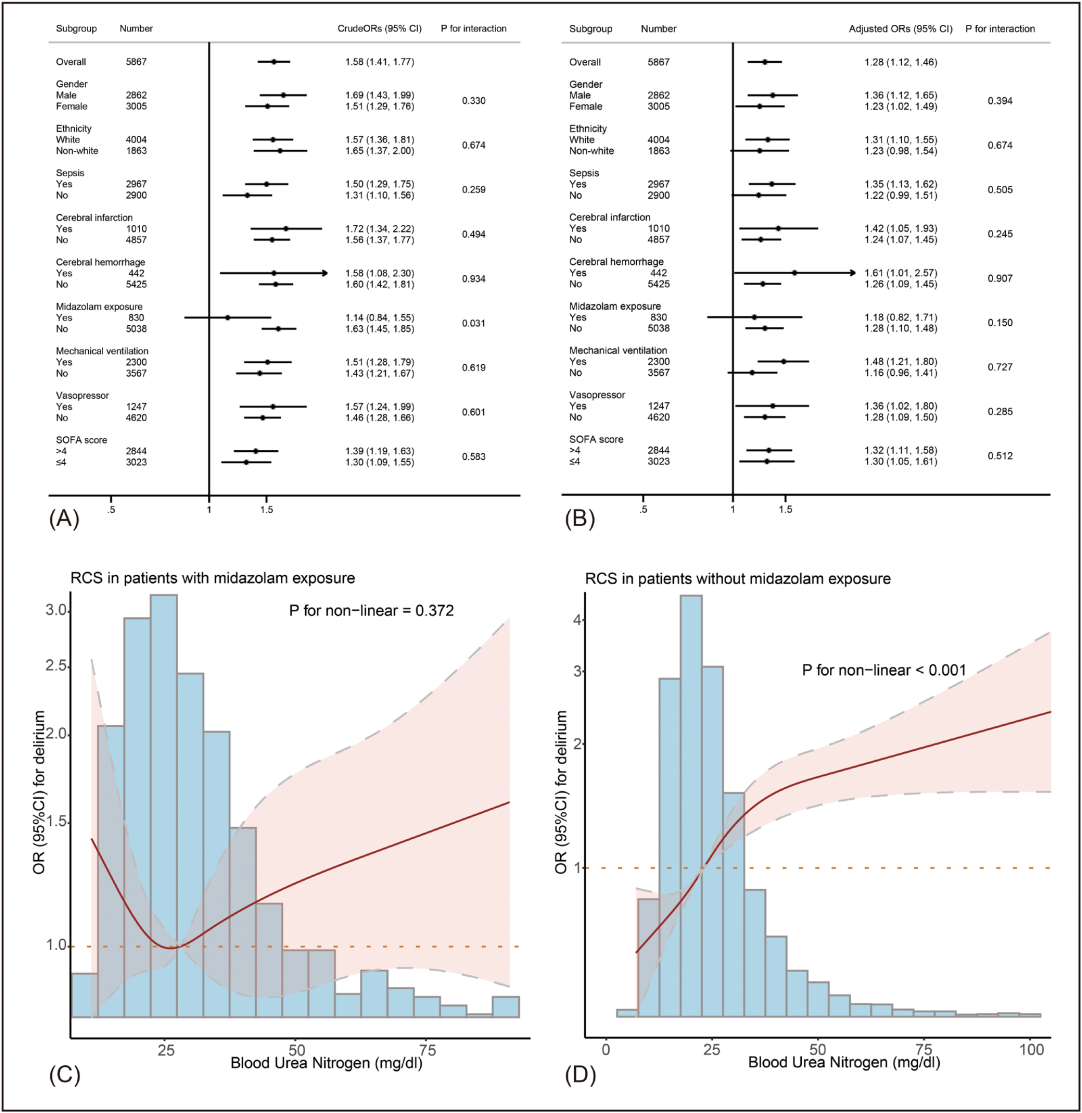
Result of subgroup analysis. (A, B) The forest plots present the crude and adjusted odds ratios of high BUN (> 23 mg/dL) on the risk of delirium using the unadjusted (A) and adjusted (B) logistic regression model 3 in Table 3. (C, D) The RCS curve visualized the association between BUN and the risk of delirium in patients with (C) and without (D) midazolam exposure.

In the subgroup analysis of the unadjusted model, a significant interaction effect between BUN and midazolam exposure was observed (*p* for interaction = 0.032, Figure 3A). However, this interaction was not significant in the adjusted model (Figure 3B). Among patients who received midazolam, there was no significant association between high BUN and the odds of delirium. In contrast, among those not exposed to midazolam, a more pronounced association between BUN and delirium was found (Figure 3C,D). No other significant interaction effects were observed (*p* for interaction > 0.05). In all other subgroups, high BUN was significantly associated with increased odds of delirium (all OR > 1). However, this association did not reach statistical significance in the adjusted models for non‐white ethnicity, sepsis (+), midazolam exposure (+), and mechanical ventilation (+) subgroups, where the 95% CI included 1.

### 3.6 Sensitivity Analysis

In the PSM cohort, baseline characteristics between the high‐BUN and low‐BUN groups were largely similar, with no statistically significant differences identified, except for white blood cell levels (Table 4). The high‐BUN group exhibited a higher incidence of delirium compared to the low‐BUN group (33.51% vs. 27.17%, *p* < 0.001). Additionally, 28‐day mortality was significantly elevated in the high‐BUN group (*p* < 0.001), although no significant differences were observed in hospital mortality and ICU mortality (*p* > 0.05). Moreover, patients in the high‐BUN group had longer hospital‐LOS and ICU‐LOS (both *p* < 0.001).

**TABLE 4:** Baseline and clinical outcomes in PSM cohorts.

Variable	Overall	BUN ≤ 23 mg/dL	BUN > 23 mg/dL	*p*
Number	3092	1546	1546	
Age (years)	76.7 (70.3, 84.0)	76.7 (70.43, 84.2)	76.7 (70.3, 83.9)	0.768
Male (%)	1554 (50.26)	778 (50.32)	776 (50.19)	0.943
Ethnicity, white (%)	2112 (68.31)	1052 (68.05)	1060 (68.56)	0.757
Weight (kg)	75.0 (62.6, 87.0)	75.0 (63.0, 86.6)	75.0 (62.2, 87.2)	0.871
Comorbidity				
Sepsis (%)	1512 (48.90)	758 (49.03)	754 (48.77)	0.886
Cerebral infarction (%)	546 (17.66)	273 (17.66)	273 (17.66)	> 0.999
Cerebral hemorrhage (%)	276 (8.93)	137 (8.86)	139 (8.99)	0.900
Coronary heart disease (%)	701 (22.67)	350 (22.64)	351 (22.70)	0.966
Heart failure (%)	731 (23.64)	376 (24.32)	355 (22.96)	0.745
Hypertension (%)	2053 (66.40)	1023 (66.17)	1030 (66.62)	0.790
Diabetes mellitus (%)	791 (25.58)	405 (26.20)	386 (24.97)	0.434
Atrial fibrillation (%)	1185 (38.332)	588 (38.03)	597 (38.62)	0.739
Anemia (%)	668 (21.60)	330 (21.35)	338 (21.86)	0.727
Chronic pulmonary disease (%)	829 (26.81)	416 (26.91)	413 (26.71)	0.903
Liver disease (%)	143 (4.62)	72 (4.66)	71 (4.59)	0.932
Malignant cancer (%)	448 (14.49)	218 (14.10)	230 (14.88)	0.540
Laboratory parameter				
White blood cell (k/μL)	10.4 (7.8, 13.7)	10.0 (7.6, 13.7)	10.5 (8.1, 13.6)	0.026
Hemoglobin (g/dL)	11.1 (9.7, 12.5)	11.1 (9.8, 12.5)	11.1 (9.6, 12.5)	0.798
Platelets (k/μL)	192 (144, 249)	192 (144, 247)	192 (144, 251)	0.904
Sodium (mmol/L)	139 (136, 141)	139 (136, 141)	138 (136, 141)	0.728
Potassium (mmol/L)	4.1 (3.7, 4.4)	4.1 (3.7, 4.5)	4.1 (3.7, 4.4)	0.973
Creatinine (mg/dL)	0.9 (0.8, 1.1)	0.9 (0.8, 1.1)	0.9 (0.8, 1.1)	0.722
Intervention				
Mechanical ventilation (%)	1255 (40.59)	637 (41.20)	618 (39.97)	0.487
Vasoactive drug (%)	590 (19.08)	298 (19.28)	292 (18.89)	0.784
Midazolam exposure (%)	421 (13.62)	214 (13.84)	207 (13.39)	0.714
Disease severity score				
SOFA score	4 (3, 6)	4 (3, 6)	4 (3, 6)	0.872
SAPS II score	36 (31, 42)	36 (30, 42)	36 (31, 42)	0.311
Outcomes				
Delirium (%)	938 (30.34)	420 (27.17)	518 (33.51)	< 0.001
Hospital mortality (%)	255 (8.25)	114 (7.37)	141 (9.12)	0.078
ICU mortality (%)	146 (4.72)	72 (4.66)	74 (4.79)	0.865
28‐day mortality (%)	409 (13.23)	174 (11.25)	235 (15.20)	0.001
90‐day mortality (%)	589 (19.05)	247 (15.98)	342 (22.12)	< 0.001
Hospital LOS (days)	7.8 (5.4, 12.1)	7.0 (5.0, 10.3)	8.8 (6.0, 13.9)	< 0.001
ICU LOS (days)	3.2 (2.5, 4.9)	3.1 (2.4, 4.3)	3.6 (2.7, 5.7)	< 0.001

*Note:* To estimate propensity scores, a multivariate logistic regression model was applied. A 1:1 nearest neighbor matching technique, without replacement, was employed, utilizing a caliper width of 0.02 to ensure precise and balanced group comparisons.

Abbreviations: BUN, blood urea nitrogen; ICU, intensive care unit; LOS, length of stay; SAPS, Simplified Acute Physiology Score; SOFA, Sequential Organ Failure Assessment; Tip: Continuous variables are presented as median (first quartile–third quartile), while categorical variables are expressed as count (percentage).

High BUN levels were significantly associated with increased odds of both delirium and 28‐day mortality in the PSM cohort (Table 5), with 35%–39% increased odds of delirium (OR = 1.35–1.39, all *p* < 0.001) and 41%–48% increased odds of 28‐day mortality (OR = 1.41–1.48, all *p* ≤ 0.001).

**TABLE 5:** Logistic regression analysis of the impact of BUN > 23 mg/dL on delirium and 28‐day mortality in elderly patients with sepsis in the PSM cohort.

	Delirium		28‐day mortality	
	OR (95% CI)	*p*	OR (95% CI)	*p*
Unadjusted model	1.35 (1.16–1.57)	< 0.001	1.41 (1.15–1.74)	0.001
Adjusted Model 1	1.39 (1.19–1.64)	< 0.001	1.48 (1.19–1.84)	< 0.001
Adjusted Model 2	1.39 (1.18–1.63)	< 0.001	1.47 (1.18–1.84)	0.001
Adjusted Model 3	1.39 (1.18–1.64)	< 0.001	1.48 (1.19–1.86)	0.001

*Note:* Model 1: adjustments were made for sex, age, ethnicity, body weight, and comorbidities, including cerebral infarction, cerebral hemorrhage, sepsis, hypertension, coronary heart disease, heart failure, atrial fibrillation, diabetes, chronic pulmonary disease, liver disease, anemia, and malignant cancer. Model 2: built upon Model 1 by adjusting for additional laboratory parameters, such as serum white blood cells, hemoglobin, platelets, sodium, potassium, and creatinine levels. Model 3: built upon Model 2 by additionally adjusting for mechanical ventilation, vasoactive drugs, SOFA score, and SAPS II score.

Abbreviations: CI, confidence interval; OR, odds ratio; SAPS II, Simplified Acute Physiology Score‐2; SOFA, Sequential Organ Failure Assessment.

### 3.7 Mendelian Randomization Analysis

**FIGURE 4:**
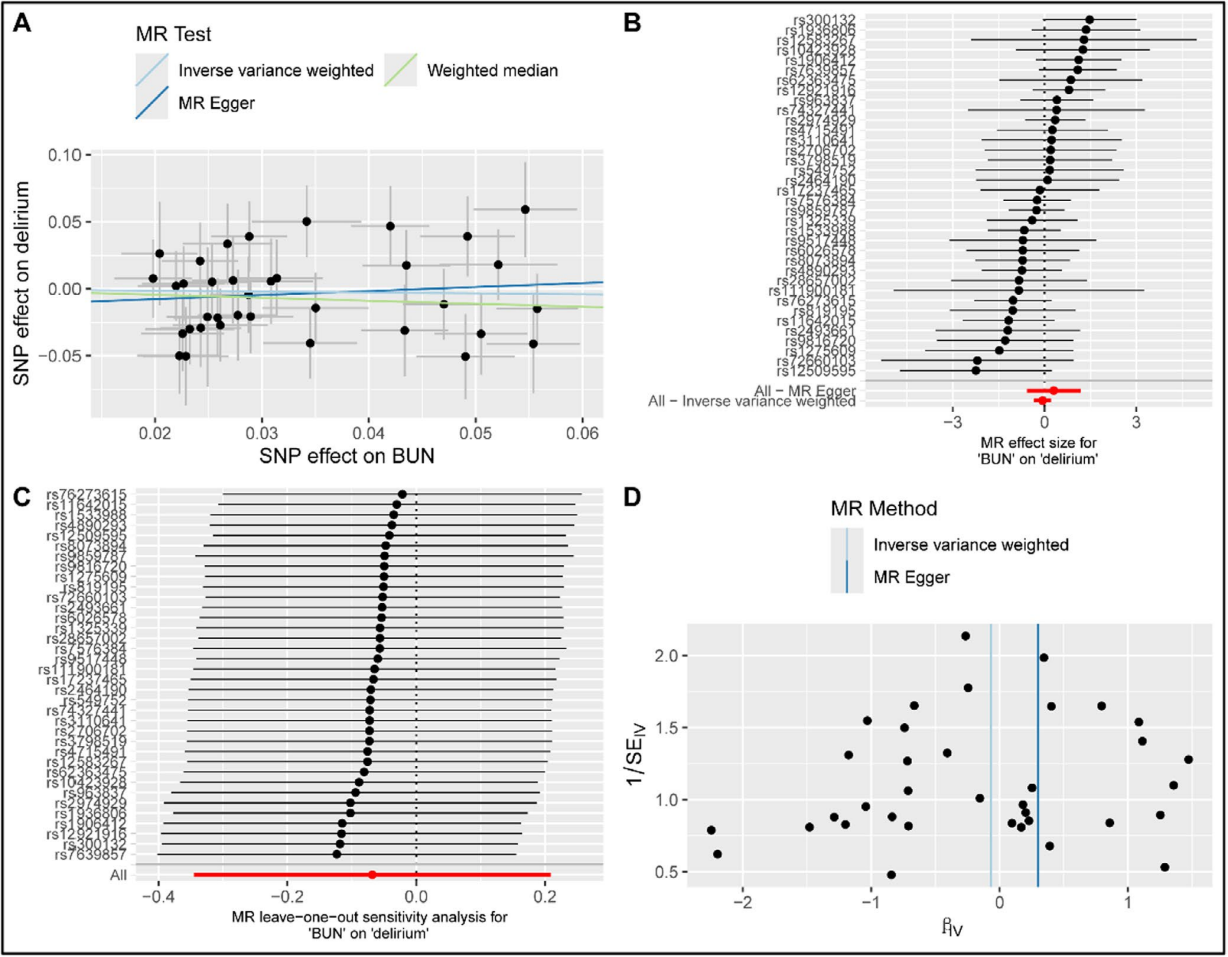
Visualization results for describing the causal association between serum BUN and the risk of developing delirium. (A) scatter plot; (B) forest plot; (C) leave‐one‐out sensitivity analysis plot; (D) funnel plot. No significant result was found in this MR analysis.

The MR analysis results indicated no causal relationship between serum BUN levels and delirium across all statistical methods (all *p* > 0.05, Table 6). The scatter plot, forest plot, leave‐one‐out sensitivity analysis plot, and funnel plot are depicted in Figure 4. MR‐Egger regression intercepts and Cochran's Q test showed no significant directional horizontal pleiotropy (*p* = 0.391) or heterogeneity (*p* = 0.409) among the IVs. Details regarding proxy SNPs are provided in Table S7.

**TABLE 6:** MR analysis results illustrating the causal relationship between BUN and delirium.

Exposure	Outcome	SNP	Method	OR (95% CI)	*p*
BUN	Delirium	36	Inverse variance weighted method under random effects	0.93 (0.71–1.23)	0.630
			Weighted median method	0.80 (0.54–1.19)	0.273
			MR‐Egger regression method	1.35 (0.56–3.24)	0.506

## 4 Discussion

This retrospective study demonstrated that elevated BUN levels are associated with an increased likelihood of delirium in critically ill elderly patients who do not have renal injury and are not receiving renal replacement therapy. The results suggest that elevated BUN may be a risk factor for delirium development, following a nonlinear positive association. However, the MR analysis did not reveal a causal relationship. Notably, the use of midazolam significantly influenced this relationship, as the association between elevated BUN and delirium was only observed in patients who had not been exposed to midazolam. In conclusion, even in the absence of renal injury or renal replacement therapy, elevated BUN remains a promising biomarker for predicting delirium. When utilizing BUN for this purpose, it is critical to account for the potential confounding effect of midazolam.

Urea is the nitrogenous byproduct of protein and amino acid metabolism, and BUN levels are commonly used to reflect the nitrogen content of urea in the blood. Since urea is primarily excreted through the kidneys, BUN is frequently used as a clinical biomarker for both acute and chronic kidney injury [28]. Previous studies have shown that abnormal BUN levels are linked to poor prognosis in various diseases, such as stroke, cerebral hemorrhage, infective endocarditis, heart failure, acute exacerbations of chronic obstructive pulmonary disease, and critically ill patients [29, 30, 31, 32, 33, 34]. Our findings align with these studies, indicating that elevated BUN is associated with increased mortality risk. Beyond mortality prediction, abnormal BUN levels have been linked to conditions such as depression [35] and the left atrial volume index [36]. Furthermore, BUN also has predictive value in the context of various encephalopathies [37, 38]. Newman et al. identified several validated risk‐stratification models for delirium, concluding that BUN is the only laboratory indicator with predictive value for delirium [37]. Similarly, Wang et al. found a close association between elevated BUN and an increased risk of sepsis‐associated encephalopathy in pediatric ICU patients [38]. Unlike previous studies, which often considered the influence of renal factors on BUN, our research controlled for these factors, highlighting that elevated BUN levels are also predictive of delirium in critically ill elderly patients without kidney injury. This suggests that BUN's effect on delirium is not solely driven by renal dysfunction, underscoring its broader predictive significance.

The mechanism by which elevated BUN contributes to the development of delirium remains unclear. In patients with renal impairment, the accumulation of neurotoxins may explain the increased risk of encephalopathy [38]. Another significant factor is intestinal azotemia, frequently observed in cases of gastrointestinal bleeding, where elevated BUN levels result from enhanced absorption of nitrogenous compounds in the intestines [39]. To minimize the influence of intestinal azotemia, patients with gastrointestinal bleeding were excluded from the present study. Causal inference through MR analysis yielded negative results, aligning with the hypothesis that BUN acts as a biomarker associated with delirium risk, rather than as a direct causal factor. However, limitations inherent to the two‐sample MR study design, which relied on public GWAS data, restricted the analysis to linear causal relationships and did not account for the non‐linear association observed between BUN and delirium. This methodological limitation may contribute to discrepancies between the retrospective study and MR analysis. Furthermore, the GWAS data used for MR analysis originated from a population different from that of MIMIC IV, likely contributing to the observed inconsistencies between these results.

The potential impact of BUN on delirium through non‐renal and non‐gastrointestinal pathways may be explained by several mechanisms. First, BUN levels may act as a biomarker for neurohormonal activity rather than solely indicating renal insufficiency [40]. Increased sympathetic nervous system stimulation and heightened renin‐angiotensin‐aldosterone system (RAAS) activity can enhance BUN reabsorption, leading to elevated levels [40]. RAAS regulation involves two axes: the ACE/AngII/AT1R axis, which promotes RAAS activity, and the ACE2/Ang(1–7)/MasR axis, which provides negative regulation [40]. Excess AngII or AT1R activation can result in pathological conditions, such as elevated pro‐inflammatory and pro‐oxidative proteins, endothelial dysfunction, disruption of the blood–brain barrier, and vasoconstriction, all contributing to encephalopathy development [41, 42]. A study by Chen et al. demonstrated that preoperative exposure to ACEI/ARB, which inhibits RAAS activity, effectively reduced the risk of postoperative delirium [43]. Similarly, Li et al. found that ACE2‐mediated downregulation of RAAS reduced inflammation, oxidative stress, and neuronal apoptosis, thus mitigating sepsis‐associated encephalopathy [44]. Second, BUN may induce delirium through vascular damage. Urea has been shown to cause endothelial cell injury by promoting mitochondrial reactive oxygen species (ROS) production, endoplasmic reticulum stress, and local inflammatory responses [45]. Urea exposure can also inhibit the proliferation of human microvascular endothelial cells by regulating endothelial‐to‐mesenchymal transition (EndMT) [46]. Third, cyanate, a dissociation product of urea, can induce carbamylation, which affects cellular function [47]. Nitric oxide (NO), a key vasodilator produced by endothelial cells, also plays a role in inhibiting inflammation and platelet aggregation [48]. Cyanate can reduce local NO levels and contribute to endothelial damage by inhibiting the expression of endothelial nitric oxide synthase (eNOS) in a time‐ and concentration‐dependent manner [49]. Additionally, carbamylated low‐density lipoprotein disrupts endothelial function by uncoupling eNOS [50], while protein carbamylation promotes cholesterol accumulation and pro‐inflammatory signaling by interacting with macrophage scavenger receptors [51]. Fourth, elevated urea levels can increase ROS production in mouse medullary cells, contributing to insulin resistance [52]. Abnormal glucose levels, a known risk factor for delirium [53], further underscore the role of insulin resistance in delirium development. Recent research has identified a non‐linear relationship between triglyceride‐glucose index (TyG), an indicator of insulin resistance, and the risk of delirium in patients with sepsis, highlighting the significant impact of insulin resistance on delirium [54]. Fifth, increased osmolality due to elevated BUN may also contribute to delirium. Abnormal serum osmolality has been linked to higher mortality rates in patients with central nervous system injuries, such as severe brain trauma and acute ischemic stroke [55, 56]. Disrupted osmolality can promote encephalopathy and brain injury by altering cell morphology, inducing cell death, and impairing barrier function [39, 57, 58]. These results provide insight into the potential mechanisms by which elevated BUN may be linked to delirium, though further basic research is necessary to substantiate these pathways.

This study offers novel insights into the early prediction of delirium in critically ill elderly patients, suggesting that elevated BUN levels may serve as a potential biomarker for heightened delirium risk, even in patients without renal injury. These insights could facilitate earlier prevention and management of delirium in this population. However, several limitations warrant caution in interpreting the results. First, as a retrospective study, this research may be influenced by confounding factors, which could affect the accuracy of the findings. Although efforts were made to minimize confounding through multivariate logistic regression, subgroup analyses, and PSM, the possibility of unmeasured or undetected confounders cannot be fully excluded. Second, while the study identified a relationship between BUN and delirium, the underlying mechanisms remain unexplored. Although MR analysis was used to assess a potential causal link from a genetic standpoint, the results were inconclusive. Further research is needed to confirm these associations and investigate the mechanistic pathways involved. Third, the generalizability of the findings may be limited by the single‐center design, as over 68% of the final cohort comprised Caucasian patients, with significantly fewer participants from other ethnic groups, particularly Asians (only 2%). Whether these findings are applicable to non‐Caucasian and Asian populations requires further investigation. Fourth, the study used BUN levels measured at a single time point as the exposure, potentially overlooking the significance of dynamic changes in BUN over time. The trajectory of BUN fluctuations may offer more valuable insights. Future research employing trajectory analysis could provide a more comprehensive understanding of BUN's role in delirium development.

## 5 Conclusions

Serum BUN levels exhibited a non‐linear positive correlation with the likelihood of delirium in critically ill elderly patients without renal impairment, suggesting that elevated BUN may serve as a risk factor for delirium development. The potential influence of midazolam on this association should also be taken into account. Further high‐quality studies are essential to validate and strengthen these findings.

## Author Contributions

**Yipeng Fang** and **Xiaohong Tang:** writing – original draft, conceptualization, data curation, formal analysis, methodology, project administration, software, validation, visualization. Ying Gao: writing – review and editing, conceptualization, data curation, formal analysis, methodology, project administration. **Hui Xie:** writing – original draft, data curation, formal analysis, methodology. **Yuehao Shen, Min Peng** and **Jie Liu:** writing – review and editing, conceptualization. **Yunfei Zhang:** writing – review and editing, formal analysis, methodology, visualization. **Yan Cui:** writing – review and editing, project administration, supervision. **Keliang Xie:** writing – review and editing, conceptualization, funding acquisition, project administration, supervision. All of the authors gave final approval of the version to be published and agreed to be accountable for all aspects of the work.

## Ethics Statement

The MIMIC IV database has been granted ethical approval by the Institutional Review Boards of the Beth Israel Deaconess Medical Center and the Massachusetts Institute of Technology.

## Consent

The requirement for informed consent was waived, given that all information in MIMIC IV is anonymized.

## Conflicts of Interest

The authors declare no conflicts of interest.

## Supporting information


Appendix S1.

## Data Availability

All datasets used during the present study are publicly available in the MIMIC‐IV database (https://mimic.physionet.org). All detailed information on structured data and statistical analysis code can be obtained through the corresponding author upon reasonable request.
