# Supplementary material for: Association Between Blood Urea Nitrogen and Delirium in Critically Ill Elderly Patients Without Kidney Diseases: A Retrospective Study and Mendelian Randomization Analysis
Source: CNS Neurosci Ther. 2025 Jan 3;31(1):e70201. doi: 10.1111/cns.70201 (PMC11702503; doi:10.1111/cns.70201)
Supplement: Supplementary file 1 — Appendix S1. [file CNS-31-e70201-s001.docx]

**Supplemental material**

**Association between blood urea nitrogen and delirium in critically ill elderly patients without kidney diseases: A retrospective study and mendelian randomization analysis**

Yipeng Fang^1,#^, Xiaohong Tang^1,#^, Ying Gao^1,#^, Hui Xie^2^, Yuehao Shen^1^, Min Peng^1^, Jie Liu^1^, Yunfei Zhang^3^, Yan Cui^1,4,*^, Keliang Xie^1,5,*^

^1^Department of Critical Care Medicine, Tianjin Medical University General Hospital, Tianjin, 300052, China.

^2^Firth Clinical College, XinXiang Medical University, Xinxiang, 453003, Henan, China.

^3^Tianjin Hospital of Tianjin University, Tianjin, 300211, China.

^4^Department of Pathogen Biology, School of Basic Medical Sciences, Tianjin Medical University, Tianjin, 300070, China.

^5^Department of Anesthesiology, Tianjin Institute of Anesthesiology, Tianjin Medical University General Hospital, Tianjin, 300052, China.

**Supplemental list**

**Table S1** Details for comorbidity definition

**Table S2** Result of Shapiro-Wilk test

**Table S3** Clinical outcome among patients in different BUN categories based on mean values

**Table S4** Clinical outcome among patients in different BUN categories based on initial values

**Table S5** Logistic regression analysis detecting predictive value of mean BUN value on delirium and 28-day mortality in critically ill elderly patients

**Table S6** Logistic regression analysis detecting predictive value of initial BUN value on delirium and 28-day mortality in critically ill elderly patients

**Table S7** Full details of optimal proxy SNPs used in the MR analysis about the casual association between BUN and delirium

**Table S1** Details for comorbidity definition

| **Complications** | **ICD codes or Standard** |
| --- | --- |
| Diabetes mellitus | 25000, 24900, 24901, 24911, 24920, 24940, 24941, 24950, 24951, 24960, 24961, 24970, 24971, 24980, 24981, 24990, 24991, 25001, 25002, 25003, 25010, 25011, 25012, 25013, 25020, 25021, 25022, 25023, 25030, 25031, 25032, 25033, 25040, 25041, 25042, 25043, 25050, 25051, 25052, 25053, 25060, 25061, 25062, 25063, 25070, 25071, 25072, 25073, 25080, 25081, 25082, 25083, 25090, 25091, 25092, 25093, E0800, E0810, E0821, E0822, E08319, E083513, E0840, E0842, E0843, E0851, E08649, E0865, E089, E0900, E0910, E0921, E0922, E09319, E0940, E0942, E0943, E0951, E0952, E09621, E09649, E0965, E098, E099, E1010, E1011, E1021, E1022, E1029, E10311, E10319, E10321, E103213, E103219, E10329, E103291, E103292, E103293, E103299, E103312, E103313, E103319, E10339, E103391, E103393, E103399, E103411, E103413, E10349, E103491, E10351, E103511, E103512, E103513, E103519, E103522, E103523, E103531, E103532, E103559, E10359, E103591, E103592, E103593, E103599, E1036, E1039, E1040, E1041, E1042, E1043, E1044, E1049, E1051, E1052, E1059, E10610, E10618, E10620, E10621, E10622, E10628, E10641, E10649, E1065, E1069, E108, E109, E1100, E1101, E1110, E1121, E1122, E1129, E11311, E11319, E11321, E113211, E113212, E113213, E113219, E11329, E113291, E113292, E113293, E113299, E11331, E113311, E113313, E113319, E11339, E113391, E113392, E113393, E113399, E11341, E113413, E113419, E11349, E113491, E113492, E113493, E113499, E11351, E113511, E113512, E113513, E113519, E113521, E113522, E113532, E113542, E113553, E11359, E113591, E113592, E113593, E113599, E1136, E1137X9, E1139, E1140, E1141, E1142, E1143, E1144, E1149, E1151, E1152, E1159, E11610, E11618, E11620, E11621, E11622, E11628, E11641, E11649, E1165, E1169, E118, E119, E1300, E1310, E1311, E1321, E1322, E1329, E13319, E1340, E1342, E1343, E1351, E1359, E13621, E13622, E13649, E1365, E1369, E138, E139 |
| Hypertension | 4019, 4011, I10 |
| Coronary heart disease | 4111, 4142, 41181, 41402, 41404, 41405, 41407, I2101, I2102, I2109, I2111, I2119, I2121, I240, I2510, I25110, I25111, I25118, I25119, I25700, I25708, I25709, I25710, I25711, I25718, I25719, I25720, I25721, I25728, I25729, I25758, I25790, I25810, I25811, I2582, T82213A, T82218A, T82855A, T82855D, T82855S, V4581, V4582, Z951, Z955, Z9861 |
| Acute or chronic heart failure | 4280, 4281, 4289, 39891, 40201, 40211, 40291, 40401, 40403, 40411, 40413, 40491, 40493, 42820, 42821, 42822, 42823, 42830, 42831, 42832, 42833, 42840, 42841, 42842, 42843, I0981, I110, I130, I132, I5020, I5021, I5022, I5023, I5030, I5031, I5032, I5033, I5040, I5041, I5042, I5043, I50810, I50811, I50812, I50813, I50814, I5082, I5083, I5084, I5089, I509, I97130, I97131 |
| Chronic kidney disease | 5851, 5852, 5853, 5854, 5855, 5859, 28521, 40300, 40301, 40310, 40311, 40390, 40391, 40400, 40401, 40403, 40410, 40411, 40413, 40490, 40491, 40492, 40493, D631, E0822, E0922, E1022, E1122, E1322, I120, I129, I130, I1310, I1311, I132, N181, N182, N183, N184, N185, N189 |
| Atrial fibrillation | 42731, I480, I481, I4811, I4819, I482, I4820, I4821, I4891 |
| Cerebral infarction | I63132, I63133, I63139, I6319, I6320, I63211, I63212, I63213, I63219, I6322, I63231, I63232, I63233, I63239, I6329, I6330, I63311, I63312, I63313, I63321, I63322, I63331, I63332, I63333, I63341, I63342, I63343, I63349, I6339, I6340, I63411, I63412, I63413, I63419, I63421, I63422, I63423, I63429, I63431, I63432, I63433, I63439, I63441, I63442, I63443, I63449, I6349, I6350, I63511, I63512, I63513, I63521, I63522, I63523, I63529, I63531, I63532, I63533, I63539, I63541, I63542, I63543, I63549, I6359, I636, I638, I6381, I6389, I639, I6930, I6931, I69310, I69311, I69312, I69313, I69315, I69318, I69319, I69320, I69321, I69322, I69323, I69328, I69331, I69333, I69334, I69341, I69344, I69351, I69352, I69353, I69354, I69359, I69364, I69365, I69369, I69390, I69391, I69392, I69393, I69398, V1254, Z8673 |
| Cerebral hemorrhage | 431, I619, I610, I611, I612, I613, I614, I615, I616, I618, I6911, I69110, I69111, I69112, I69118, I69119, I69120, I69121, I69122, I69128, I69131, I69132, I69134, I69141, I69144, I69151, I69152, I69153, I69154, I69159, I69164, I69165, I69169, I69190, I69191, I69192, I69193, I69198 |
| Gastrointestinal bleeding | 4560, 45620, 53021, I8501, I8511, K2211, K2901, K2921, K2941, K2951, K2961, K2971, K2981, K2991, K31811, K5701, K5711, K5713, K5721, K5731, K5733, K5751, K5791, K5793, |
| Anemia | 2819, 2800, 2801, 2808, 2809, 2810, 2811, 2812, 2813, 2823, 2828, 2829, 2830, 2839, 2849, 2850, 2851, 2853, 2858, 28310, 28319, 28409, 28489, D461, D4620, D4621, D4622, D464, D500, D508, D509, D510, D511, D513, D518, D519, D520, D521, D528, D529, D531, D538, D539, D588, D589, D590, D591, D592, D594, D598, D599, D6109, D611, D612, D613, D6189, D619, D62, D641, D643, D644, D6489, D75A, O368230 |
| Sepsis | According to the Sepsis 3 structured view |
| Chronic pulmonary disease | According to the Charlson structured view |
| Liver disease | According to the Charlson structured view |
| Malignant cancer | According to the Charlson structured view |

**Table S2** Result of Shapiro-Wilk test

|  | ***P*-value** | **Distribution** |
| --- | --- | --- |
| Age (years) | <0.001 | Skewed Distribution |
| Weight (kg) | <0.001 | Skewed Distribution |
| White blood cell (k/uL) | <0.001 | Skewed Distribution |
| Hemoglobin (g/dL) | 0.038 | Skewed Distribution |
| Platelets (k/uL) | <0.001 | Skewed Distribution |
| Sodium (mmol/L) | <0.001 | Skewed Distribution |
| Potassium (mmol/L) | <0.001 | Skewed Distribution |
| Creatinine (mg/dL) | <0.001 | Skewed Distribution |
| Mean blood pressure (mmHg) | <0.001 | Skewed Distribution |
| Blood urea nitrogen (mg/dL) |  | Skewed Distribution |
| Initial value | <0.001 | Skewed Distribution |
| Maximum value | <0.001 | Skewed Distribution |
| Mean value | <0.001 | Skewed Distribution |
| Hospital LOS (days) | <0.001 | Skewed Distribution |
| ICU LOS (days) | <0.001 | Skewed Distribution |

**Table S3** Clinical outcome among patients in different BUN categories based on mean values

|  | **Q1(BUN≤13.5mg/dL)** | **Q2(13.5<BUN≤17.6mg/dL)** | **Q3(17.6<BUN≤23.0mg/dL)** | **Q4(BUN>23.0mg/dL)** | ***P* value** |
| --- | --- | --- | --- | --- | --- |
| Number (%) | 1471(25.07) | 1459(24.87) | 1482(25.26) | 1455(24.80) |  |
| Delirium (%) | 402(27.33) | 413(28.31) | 448(30.23) | 485(33.33) | 0.002 |
| 28-day mortality (%) | 117(7.95) | 157(10.76) | 184(12.42) | 318(21.86) | <0.001 |
| 90-day mortality (%) | 193(13.12) | 211(14.46) | 266(17.95) | 463(31.82) | <0.001 |
| hospital mortality (%) | 73(4.96) | 93(6.37) | 115(7.76) | 223(15.33) | <0.001 |
| ICU mortality (%) | 46(3.13) | 53(3.63) | 73(4.93) | 111(7.63) | <0.001 |
| Hospital LOS (days) | 7.4(5.1,11.1) | 7.5(5.2,11.8) | 7.8(5.5,11.9) | 8.4(5.7,13.1) | <0.001 |
| ICU LOS (days) | 3.1(2.4,4.3) | 3.2(2.4,4.7) | 3.2(2.5,4.9) | 3.5(2.6,5.4) | <0.001 |

a. Continuous variables are displayed as mean (standard deviation) or median (first quartile–third quartile); categorical variables are displayed as count (percentage); ICU, intensive care unit; LOS, length of stays; BUN, blood urea nitrogen.

**Table S4** Clinical outcome among patients in different BUN categories based on initial values

|  | **Q1(BUN≤13mg/dL)** | **Q2(13<BUN≤18mg/dL)** | **Q3(18<BUN≤24mg/dL)** | **Q4(BUN>24mg/dL)** | ***P* value** |
| --- | --- | --- | --- | --- | --- |
| Number (%) | 1546(26.35) | 1696(28.91) | 1221(20.81) | 1404(23.93) |  |
| Delirium (%) | 448(28.98) | 455(26.83) | 373(30.55) | 472(33.62) | <0.001 |
| 28-day mortality (%) | 140(9.06) | 195(11.50) | 168(13.76) | 273(19.44) | <0.001 |
| 90-day mortality (%) | 205(13.26) | 284(16.75) | 247(20.23) | 397(28.28) | <0.001 |
| hospital mortality (%) | 89(5.76) | 126(7.43) | 105(8.60) | 184(13.11) | <0.001 |
| ICU mortality (%) | 56(3.62) | 74(4.36) | 56(4.59) | 97(6.91) | <0.001 |
| Hospital LOS (days) | 7.7(5.3,12.0) | 7.5(5.1,11.4) | 7.8(5.5,11.9) | 8.1(5.6,12.8) | <0.001 |
| ICU LOS (days) | 3.2(2.4,4.7) | 3.2(2.4,4.7) | 3.3(2.6,5.0) | 3.2(2.6,4.8) | 0.004 |

a. Continuous variables are displayed as mean (standard deviation) or median (first quartile–third quartile); categorical variables are displayed as count (percentage); ICU, intensive care unit; LOS, length of stays; BUN, blood urea nitrogen.

**Table S5** Logistic regression analysis detecting predictive value of mean BUN value on delirium and 28-day mortality in critically ill elderly patients

|  | **Unadjusted** | | **Model 1** | | **Model 2** | | **Model 3** | |
| --- | --- | --- | --- | --- | --- | --- | --- | --- |
|  | **OR (95%CI)** | ***P* value** | **OR (95%CI)** | ***P* value** | **OR (95%CI)** | ***P* value** | **OR (95%CI)** | ***P* value** |
| Delirium | | | | | | | | |
| BUN (mg/dL) | 1.01(1.01-1.02) | <0.001 | 1.00(1.01-1.02) | 0.003 | 1.01(1.00-1.02) | 0.022 | 1.00(1.00-1.01) | 0.246 |
| Q1(BUN≤13.5mg/dL) | Reference | | | | | | | |
| Q2(13.5<BUN≤17.6mg/dL) | 1.05(0.89-1.23) | 0.554 | 1.01(0.85-1.19) | 0.953 | 0.99(0.54-1.32) | 0.925 | 0.92(0.77-1.09) | 0.330 |
| Q3(17.6<BUN≤23.0mg/dL) | 1.15(0.98-1.35) | 0.082 | 1.14(0.96-1.35) | 0.132 | 1.09(0.92-1.30) | 0.320 | 1.00(0.84-1.19) | 0.985 |
| Q4(BUN>23.0mg/dL) | 1.33(1.14-1.56) | <0.001 | 1.18(1.00-1.41) | 0.054 | 1.12(0.92-1.36) | 0.252 | 0.97(0.79-1.18) | 0.744 |
| 28-day mortality | | | | | | | | |
| BUN (mg/dL) | 1.05(1.04-1.06) | <0.001 | 1.05(1.04-1.06) | <0.001 | 1.07(1.06-1.09) | <0.001 | 1.07(1.06-1.08) | <0.001 |
| Q1(BUN≤13.5mg/dL) | Reference | | | | | | | |
| Q2(13.5<BUN≤17.6mg/dL) | 1.40(1.09-1.79) | 0.009 | 1.40(1.08-1.81) | 0.012 | 1.47(1.13-1.90) | 0.004 | 1.37(1.05-1.79) | 0.022 |
| Q3(17.6<BUN≤23.0mg/dL) | 1.64(1.29-2.09) | <0.001 | 1.70(1.31-2.19) | 0.001 | 1.85(1.42-2.40) | <0.001 | 1.68(1.29-2.19) | <0.001 |
| Q4(BUN>23.0mg/dL) | 3.24(2.58-4.06) | <0.001 | 3.23(2.52-4.13) | <0.001 | 3.91(2.99-5.12) | <0.001 | 3.37(2.56-4.43) | <0.001 |

Model 1 = adjusting sex, age, ethnicity, body weight and comorbidities (cerebral infarction, cerebral hemorrhage, sepsis, hypertension, coronary heart disease, heart failure, atrial fibrillation, diabetes, chronic pulmonary disease, liver disease, anemia, malignant cancer).

Model 2 = Model 1 + adjusting laboratory parameters (serum white blood cells, hemoglobin, platelet, sodium, potassium and creatinine)

Model 3 = Model 2 + mechanical ventilation, usage of vasoactive drugs, midazolam exposure, SOFA score and SAPSII score

CI: confidence interval; OR: odds ratio; SOFA, Sequential Organ Failure Assessment, SAPSII, Simplified Acute Physiology Score-2

**Table S6** Logistic regression analysis detecting predictive value of initial BUN value on delirium and 28-day mortality in critically ill elderly patients

|  | **Unadjusted** | | **Model 1** | | **Model 2** | | **Model 3** | |
| --- | --- | --- | --- | --- | --- | --- | --- | --- |
|  | **OR (95%CI)** | ***P* value** | **OR (95%CI)** | ***P* value** | **OR (95%CI)** | ***P* value** | **OR (95%CI)** | ***P* value** |
| Delirium | | | | | | | | |
| BUN (mg/dL) | 1.01(1.01-1.01) | <0.001 | 1.01(1.00-1.01) | 0.012 | 1.01(1.00-1.01) | 0.035 | 1.00(1.00-1.01) | 0.203 |
| Q1(BUN≤13mg/dL) | Reference | | | | | | | |
| Q2(13<BUN≤18mg/dL) | 0.90(0.77-1.05) | 0.173 | 0.89(0.75-1.04) | 0.141 | 0.87(0.74-1.03) | 0.105 | 0.87(0.74-1.03) | 0.105 |
| Q3(18<BUN≤24mg/dL) | 1.08(0.91-1.27) | 0.369 | 1.09(0.91-1.29) | 0.343 | 1.06(0.89-1.26) | 0.526 | 1.06(0.88-1.27) | 0.525 |
| Q4(BUN>24mg/dL) | 1.24(1.06-1.45) | 0.007 | 1.11(0.94-1.32) | 0.214 | 1.08(0.89-1.31) | 0.436 | 1.02(0.84-1.25) | 0.823 |
| 28-day mortality | | | | | | | | |
| BUN (mg/dL) | 1.02(1.01-1.02) | <0.001 | 1.02(1.01-1.02) | <0.001 | 1.02(1.01-1.03) | <0.001 | 1.02(1.01-1.02) | <0.001 |
| Q1(BUN≤13mg/dL) | Reference | | | | | | | |
| Q2(13<BUN≤18mg/dL) | 1.30(1.04-1.64) | 0.023 | 1.30(1.02-1.65) | 0.033 | 1.34(1.05-1.70) | 0.018 | 1.30(1.02-1.67) | 0.034 |
| Q3(18<BUN≤24mg/dL) | 1.60(1.26-2.03) | <0.001 | 1.58(1.23-2.03) | <0.001 | 1.64(1.27-2.12) | <0.001 | 1.57(1.21-2.03) | 0.001 |
| Q4(BUN>24mg/dL) | 2.42(1.95-3.02) | <0.001 | 2.05(1.90-3.06) | <0.001 | 2.64(2.03-3.44) | <0.001 | 2.34(1.78-3.07) | <0.001 |

Model 1 = adjusting sex, age, ethnicity, body weight and comorbidities (cerebral infarction, cerebral hemorrhage, sepsis, hypertension, coronary heart disease, heart failure, atrial fibrillation, diabetes, chronic pulmonary disease, liver disease, anemia, malignant cancer).

Model 2 = Model 1 + adjusting laboratory parameters (serum white blood cells, hemoglobin, platelet, sodium, potassium and creatinine)

Model 3 = Model 2 + mechanical ventilation, usage of vasoactive drugs, midazolam exposure, SOFA score and SAPSII score

CI: confidence interval; OR: odds ratio; SOFA, Sequential Organ Failure Assessment, SAPSII, Simplified Acute Physiology Score-2

**Table S7** Full details of optimal proxy SNPs used in the MR analysis about the casual association between BUN and delirium

| **Exposure** | **SNP** | **effect_allele.exposure** | **other_allele.exposure** | **beta.exposure** | **se.exposure** | **Fvalue** | **pval.exposure** | **beta.outcome** | **se.outcome** | **pval.outcome** |
| --- | --- | --- | --- | --- | --- | --- | --- | --- | --- | --- |
| BUN | rs10423928 | A | T | -0.02677 | 0.004197 | 40.68355 | 1.80E-10 | -0.03352 | 0.029935 | 0.262835 |
| BUN | rs111900181 | G | A | 0.0249 | 0.00382 | 42.48856 | 7.15E-11 | -0.021 | 0.052001 | 0.686396 |
| BUN | rs11642015 | T | C | 0.03452 | 0.004394 | 61.71937 | 4.01E-15 | -0.04061 | 0.026344 | 0.123155 |
| BUN | rs12509595 | C | T | -0.02227 | 0.003938 | 31.98078 | 1.55E-08 | 0.050008 | 0.028227 | 0.076447 |
| BUN | rs12583267 | G | A | -0.02042 | 0.003554 | 33.01236 | 9.26E-09 | -0.02628 | 0.03837 | 0.493442 |
| BUN | rs1275609 | A | G | -0.02257 | 0.003873 | 33.96003 | 5.61E-09 | 0.033419 | 0.027859 | 0.230303 |
| BUN | rs12921916 | C | T | -0.04923 | 0.004414 | 124.3927 | 6.91E-29 | -0.03912 | 0.029825 | 0.189661 |
| BUN | rs1325339 | C | T | -0.03503 | 0.004982 | 49.43936 | 2.03E-12 | 0.014278 | 0.026443 | 0.589233 |
| BUN | rs1533988 | T | A | 0.0505 | 0.004331 | 135.9585 | 2.08E-31 | -0.03359 | 0.030546 | 0.271499 |
| BUN | rs17237465 | C | T | 0.02874 | 0.003598 | 63.80449 | 1.37E-15 | -0.00443 | 0.028448 | 0.876252 |
| BUN | rs1906412 | C | G | -0.04201 | 0.003661 | 131.6758 | 1.78E-30 | -0.04672 | 0.029863 | 0.117744 |
| BUN | rs1936806 | T | C | 0.02881 | 0.003544 | 66.08442 | 4.31E-16 | 0.039053 | 0.026181 | 0.13579 |
| BUN | rs2464190 | C | T | 0.02196 | 0.00359 | 37.41759 | 9.54E-10 | 0.002106 | 0.026226 | 0.935992 |
| BUN | rs2493661 | T | C | -0.02428 | 0.003795 | 40.93302 | 1.57E-10 | 0.029167 | 0.029301 | 0.319531 |
| BUN | rs2706702 | T | C | -0.02534 | 0.003548 | 51.0089 | 9.11E-13 | -0.00515 | 0.027805 | 0.853199 |
| BUN | rs28657002 | A | T | 0.02585 | 0.004124 | 39.29016 | 3.69E-10 | -0.02164 | 0.029315 | 0.460369 |
| BUN | rs2974929 | C | T | -0.05212 | 0.005525 | 88.99062 | 3.99E-21 | -0.01799 | 0.026245 | 0.493027 |
| BUN | rs300132 | C | G | 0.03418 | 0.005134 | 44.32333 | 2.79E-11 | 0.05031 | 0.026711 | 0.059636 |
| BUN | rs3110641 | G | A | -0.0273 | 0.003842 | 50.4906 | 1.19E-12 | -0.00624 | 0.031971 | 0.845334 |
| BUN | rs3798519 | C | A | 0.03085 | 0.003923 | 61.8406 | 3.75E-15 | 0.00559 | 0.031922 | 0.860991 |
| BUN | rs4715491 | G | A | 0.03139 | 0.004306 | 53.14159 | 3.11E-13 | 0.007932 | 0.02899 | 0.784381 |
| BUN | rs4890293 | G | A | 0.05539 | 0.004361 | 161.3209 | 5.77E-37 | -0.04102 | 0.036937 | 0.266809 |
| BUN | rs549752 | G | A | -0.02266 | 0.003888 | 33.96779 | 5.62E-09 | -0.00382 | 0.027996 | 0.891531 |
| BUN | rs6026578 | G | C | -0.02895 | 0.003967 | 53.25651 | 2.94E-13 | 0.02065 | 0.027252 | 0.448608 |
| BUN | rs62363475 | T | C | 0.02421 | 0.003554 | 46.40393 | 9.59E-12 | 0.020755 | 0.028823 | 0.471461 |
| BUN | rs72660103 | T | C | -0.02288 | 0.003982 | 33.01487 | 9.16E-09 | 0.050269 | 0.036758 | 0.171453 |
| BUN | rs74327441 | A | G | -0.01981 | 0.003618 | 29.98001 | 4.39E-08 | -0.00773 | 0.029168 | 0.790934 |
| BUN | rs7576384 | G | C | 0.04703 | 0.003828 | 150.9406 | 1.06E-34 | -0.01155 | 0.026469 | 0.662686 |
| BUN | rs76273615 | G | A | 0.04904 | 0.004612 | 113.0635 | 2.11E-26 | -0.05054 | 0.031658 | 0.11039 |
| BUN | rs7639857 | C | G | 0.05464 | 0.00484 | 127.4473 | 1.48E-29 | 0.059235 | 0.0355 | 0.095194 |
| BUN | rs8073894 | C | T | 0.04336 | 0.004082 | 112.8321 | 2.35E-26 | -0.03115 | 0.034174 | 0.362068 |
| BUN | rs819195 | C | T | -0.0261 | 0.004446 | 34.46213 | 4.34E-09 | 0.027212 | 0.02739 | 0.320452 |
| BUN | rs9517448 | C | A | 0.02774 | 0.003736 | 55.13142 | 1.12E-13 | -0.01971 | 0.033913 | 0.561204 |
| BUN | rs963837 | C | T | -0.04352 | 0.004096 | 112.8906 | 2.24E-26 | -0.01755 | 0.026408 | 0.50642 |
| BUN | rs9816720 | A | G | 0.02324 | 0.004185 | 30.83765 | 2.83E-08 | -0.02995 | 0.026417 | 0.256916 |
| BUN | rs9859787 | G | A | -0.05576 | 0.003809 | 214.3007 | 1.60E-48 | 0.014804 | 0.026099 | 0.570553 |
